# Desmocollin-3 and Bladder Cancer

**DOI:** 10.3390/diseases13050131

**Published:** 2025-04-23

**Authors:** Chandreshwar P. Shukla, Nayan K. Jain, Michael A. O’Donnell, Kapil V. Vachhani, Rashmi Patel, Janki Patel, Rajiv Modi, Arpit Dheeraj, Jee Min Lee, Annah Rolig, Sanjay V. Malhotra, Bakulesh Khamar

**Affiliations:** 1 Research and Development, Auro Vaccines Pvt Ltd., Hyderabad 502329, India; 2 Life Science Department, Gujarat University, Navrangpura 380009, Ahmedabad, India; 3 Department of Urology, University of Iowa Carver College of Medicine, Iowa City, IA 52242, USA; 4 Cadila Pharmaceuticals Ltd., Trasad Road, Dholka 382225, Ahmedabad, India; 5 Institute of Kidney Diseases and Research Centre, Institute of Transplantation Sciences Civil Hospital Campus, Asarwa, Ahmedabad 380016, India; 6 Department of Pathology, Pandit Deendayal Upadhyay Medical Collage, Rajkot 360001, India; 7 Department of Cell, Development and Cancer Biology, Knight Cancer Institute, Oregon Health & Science University, Portland, OR 97201, USAleejee@ohsu.edu (J.M.L.);

**Keywords:** desmocollin-3, immune infiltrates, bladder cancer

## Abstract

Background: Desmocollin3, a transmembrane protein, is expressed in the basal/suprabasal layer of normal stratified epithelium. DSC3 gene expression is described in muscle-invasive bladder cancer (MIBC). DSC3-protein-expressing recurrent non-muscle-invasive bladder cancer (NMIBC) had a durable response to CADI-03, a DSC3-specific active immunotherapy. Methods: We evaluated DSC3 protein expression and its correlation with tumor-infiltrating immune cells in bladder cancer. DSC3 gene expression and its correlation with 208 immune encoding genes, treatment outcome, and survival were evaluated using the “ARRAYEXPRESS” and “TCGA” datasets. Immune genes were grouped as tumor-controlling immune genes (TCIGs) and tumor-promoting immune genes (TPIGs) as per their functions. Results & conclusions: NMIBC had higher DSC3 expression compared to MIBC. More immune genes were correlated with DSC3 in MIBC (21) compared to NMIBC (11). Amongst the TCIGs, six in NMIBC and one in MIBC had a negative correlation while two in NMIBC and nine in MIBC had a positive correlation with DSC3. Amongst the TPIGs, nine in NMIBC and five in MIBC had a negative correlation. Seven TPIGs had a positive correlation with DSC3 in MIBC and none in NMIBC. Of the T cell exhaustion markers, none were correlated with DSC3 in MIBC. Among NMIBC, CTLA4 and TIGIT were the only markers of exhaustion that demonstrated a negative correlation with DSC3. DSC3 expression was also higher in p53 mutant compared to wild p53, non-papillary MIBC compared to papillary MIBC, and in basal, squamous molecular subtype compared to luminal MIBC. MIBC with lower DSC3 expression had better outcomes (response, survival) compared to those with higher DSC3 expression.

## 1. Introduction

Desmocollin-3 (DSC3) is one of the desmosomal cell-adhesion molecules (desmogleins 1-4 and desmocollin 1-3) that helps maintain cell–cell adhesion [1] and participates in cell signaling as a receptor as well as a ligand [2]. It is associated with the differentiation of epithelium and not expressed by simple (single-layer) epithelium. It is present as a membranous protein (on the surface of cells) in the basal/suprabasal layer of the normal stratified epithelium of organs like the tongue, buccal mucosa, esophagus, vagina, etc. [2]. Its cytoplasmic expression is known in rapidly dividing cells as seen during embryogenesis. DSC3 is produced by the endoplasmic reticulum (ER) and transported to the plasma membrane. ER stress is associated with aberrant expression of DSC3. Aberrant DSC3 expression includes cytoplasmic expression alone, cytoplasmic expression with membranous expression, or total loss of its expression. DSC3 is a calcium-dependent transmembrane protein. Low calcium concentration is associated with ER stress, leading to aberrant expression of DSC3. Restoration of calcium concentration restores normal DSC3 expression [3]. DSC3 is a p53-responsive gene. p53 mutation is known to create a loss of DSC3 protein expression, and restoration of P53 restores DSCS3 protein expression [4]. Cancer development and progression are associated with either loss of DSC3 expression, as seen in prostate or breast cancer, or overexpression of DSC3 including its cytoplasmic expression as seen with squamous non-small-cell lung cancer and colorectal cancer [2,4]. The DSC3 gene was first cloned from bladder cancer and was found to be higher in bladder cancer tissues compared to normal bladder tissue [2]. It is described as a squamous differentiation marker in bladder cancer [5] and is seen in the basal/squamous (Ba/Sq) type of bladder cancer [5,6,7,8,9]. DSC3 protein expression is not described in MIBC. Earlier, we found the DSC3 protein expression in recurrent non-muscle-invasive bladder cancer (NMIBC) samples [10]. The relationship between DSC3 and tumor-infiltrating immune cells, treatment outcome, and survival is also not described.

In a recurrent NMIBC expressing DSC3 protein, a durable response following intradermal injection of CADI-05 has been described by us [10]. CADI-5 is an active immunotherapy that generates a DSC3-specific cell-mediated immune response [10]. The immune response after the administration of CADI-05 is linked to an enhancement in tumor immune infiltrate (TII) and a shift in the tumor immune microenvironment from immunosuppressive (decrease in FoxP3, PD-1, and CTLA4-expressing immune cells) to immunostimulatory (increase in activated CD8+T cells, macrophages, and NK cells) [11,12]. The tumor-suppressive effect of CADI-05 requires the presence of CD8+T cells, IFNγ, and IFN-α [12,13]. CADI-05 contains heat-killed mycobacterium w, a rapidly growing non-pathogenic mycobacterium. It is also known as *Mycobacterium indicus pranii* [11,12,13]. It has been approved in India for the treatment of non-small-cell lung cancer along with chemotherapy.

We evaluated DSC3 protein expression in bladder cancer and its relationship with the stage of disease as well as with tumor immune infiltrate (TII) to identify the potential of using CADI-05 in the treatment of bladder cancer. We also analyzed TCGA and other datasets for muscle-invasive bladder cancer (MIBC) and NMIBC, respectively, for the characterization of DSC3-correlated TII, the relationship of DSC3 with pathologic and molecular subtype of bladder cancer, and the impact of DSC3 expression on treatment outcome and survival to identify potential unmet medical needs in the treatment of bladder cancer.

## 2. Materials and Methods

Evaluation of DSC3 protein expression by immunohistochemistry (IHC): DSC3 protein expression was evaluated using IHC on biopsy samples from 71 patients (NMIBC = 56; MIBC = 15) participating in clinical trials [ClinicalTrials.gov: NCT00694798; https://clinicaltrials.gov/ct2/show/NCT00694798 and NCT00694915; https://clinicaltrials.gov/ct2/show/NCT00694915] (accessed on 12 June 2024) as described previously [10]. These studies were conducted following approval by their respective institutional ethics committees: the US FDA and The Central Drugs Standard Control Organization (CDSCO, the Indian regulator equivalent of US FDA). All participants provided informed consent for participation in the study. Briefly, the slides containing the tissue sections were de-paraffinized. Antigen retrieval was carried out in Tris-EDTA buffer (pH—9.0). After blocking with 3% hydrogen peroxide, the sections were incubated with mouse anti-DSC3 monoclonal antibody (Progen, Heidelberg, Germany Cat#61093 RRID: AB_2941298, dilution: 1:50). This was followed by incubation with horseradish peroxidase (HRP)-conjugated secondary antibody. The antigen–antibody reaction was visualized using DAB as the chromogen substrate. The tissue sections were then counterstained with haematoxylin. The slides were dehydrated, mounted, and observed under microscopy. Negative controls consisted of simultaneously incubated sections without primary antibodies.

**Scoring of DSC3 expression:** The DSC3 immunostaining was analyzed qualitatively by two independent pathologists. The samples with no staining/immunoreactivity for DSC3 were considered as negative while the rest were considered as positive.

**Immune infiltrates evaluation:** Haematoxylin and eosin (H&E) stained slides were evaluated by light microscopy to identify immune cells (macrophages or lymphocytes) and their localization (intrastromal or intratumoral) as previously described [14].

**In silico dataset:** An RSEM (RNAseq by expectation-maximization) of 208 genes (Supplementary Table S1) including DSC3 expression was evaluated by mRNA levels [15] in MIBC using the TCGA database (2017) The cell cancer study, available at cBioPortal.org, RRID:SCR_014555 for Cancer Genomics (https://www.cbioportal.org/, accessed on 12 June 2024 (cBioPortal, RRID:SCR_014555)) [16] as well as a supplement to the publication [6]. An FPKM (fragments per kilobase of transcript per million mapped reads) of 208 genes including DSC3 was also evaluated in early-stage urothelial carcinoma using the E-MTAB-4321 Array Express database EMTAB-4321 (https://www.ebi.ac.uk/arrayexpress/experiments/EMTAB-4321/, accessed on 12 June 2024 (EuropeanBioinformatics Institute, RRID:SCR_004727)) for evaluating spearman correlation of DSC3 gene expression and immune genes (Supplementary Table S2).

For MIBC, information about methylation status (HM450), pathological subtype, and molecular markers available with the TCGA 2017 dataset was used.

### 2.1. Bioinformatics Analysis

The transcriptome profiling data (STAR-Counts) of TCGA-BLCA dataset was downloaded, pre-processed (cor.cut = 0.6), normalized (method = “geneLength”), filtered (method = “quantile”, qnt.cut = 0.25) and analyzed using the TCGAbiolinks pipeline [17,18,19]. 406 patient BLCA samples were subdivided into two groups based on DSC3 expression (Upper and lower quartiles). Survival analysis was performed using the survminer R package [20]. Enrichment analysis was performed on genes with Spearman’s correlation ≥ 0.6 or ≤−0.6 to DSC3 expression identified on cBioportal using the TCGAbiolinks package [19].

### 2.2. Statistical Analysis

Statistical analysis for the correlation of DSC3 protein expression with clinical pathology of cancer and immune cells infiltration was assessed by χ^2^ test to estimate the *p* value, odds ratio, and its 95% confidence interval (CI) estimate using MedCalc (MedCalc RRID:SCR_015044) statistical software [https://www.medcalc.org/calc/odds_ratio.php, accessed on 12 June 2024]. Mean, median, and quartiles were calculated for RSEM and gene methylation by using Microsoft Excel (Microsoft Excel, RRID:SCR_016137) (https://www.microsoft.com/en-gb/, accessed on 12 June 2024) functions. Student’s t test was performed using GraphPad Prism (RRID:SCR_002798).

The Spearman correlation of DSC3 gene expression with other genes and immune subsets was calculated by using the online statistical tool (https://www.socscistatistics.com/tests/spearman/default2.aspx, accessed on 12 June 2024) [21]. Correlation was considered strong (0.8 or more), moderate (0.6 to 0.7), or low (0.3 to 0.5). The heat map data of MIBC samples were extracted from cBioPortal [16].

KM plotter was used for the evaluation of median overall survival (OS), hazard ratio (HR), and log-rank *p* values.

## 3. Results

### 3.1. DSC3 Expression in Bladder Cancer

Using IHC, we observed DSC3 protein expression in 39 samples out of 71 (Table 1) with a trend toward DSC3 expression being more common in NMIBC compared to MIBC [60% vs. 37.5%; *p* = 0.11] (Table 1). The proportion of tumor cells expressing DSC3 ranged from 5% to 75%.

The DSC3 gene expression followed the pattern of protein expression. Gene expression was also higher in NMIBC than in MIBC [mean FPKM value 16.1 vs. 6.3; *p* = 0.001; ArrayExpress].

**Figure 1:**
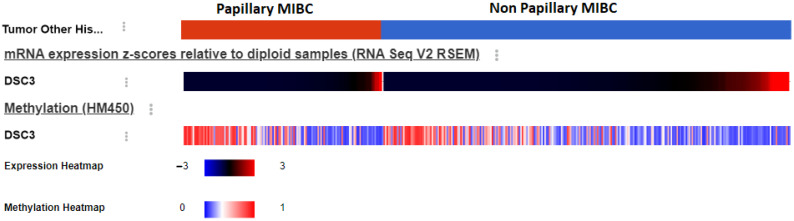
Heat map of DSC3 RSEM and its methylation.

Methylation of genes is associated with loss of DSC3 protein expression, the TCGA dataset includes information about methylation of genes obtained using Infinium HumanMethylation450 BeadChip. It provides information about the methylation status of 485,577 CpG sites. DSC3 gene expression was found to have a negative correlation (−0.7 Spearman; Figure 1), with its methylation status suggesting tumors with higher DSC3 gene expression are less likely to be methylated and more likely to express DSC3 protein.

### 3.2. DSC3 Expression and Tumor Immune Infiltrate in Bladder Cancer

DSC3 protein expression was positively associated with tumor-infiltrating immune cells (TIL) (OR 5.663; 95% CI 1.98 to 16.17; *p* = 0.001). The odds ratio for the association between DSC3 protein expression and TIL was higher for MIBC (OR 27.85; 95% CI 1.20 to 646.11) compared to NMIBC (OR 3.75; 95% CI 1.18 to 11.92) (Table 2).

Bladder cancer samples that expressed DSC3 were more likely to have intratumoral TIL than stromal TIL. (OR 15.47; *p* = 0.000 vs. OR 5.663, *p* = 0.001) (Table 3).

Irrespective of DSC3 expression, macrophages were seen in nearly all samples. There was no relationship between DSC3 expression and the occurrence of intratumoral or stromal macrophages (Table 4 and Table 5).

The TCGA dataset also provides information about the presence/absence of lymphocytes in MIBC. Lymphocytes were present in 236 (59.3%) and absent in 162 (40.7%) MIBC samples. DSC3 gene expression (Mean ± SD RSEM values) was identical in a group with lymphocytes and in a group not having lymphocytes (3403 ± 6830.9 and 3449.35 ± 8414.45; *p* = 0.4763).

### 3.3. DSC3 Gene Correlated Tumor-Infiltrating Immune Cell Signature Genes

**Figure 2:**
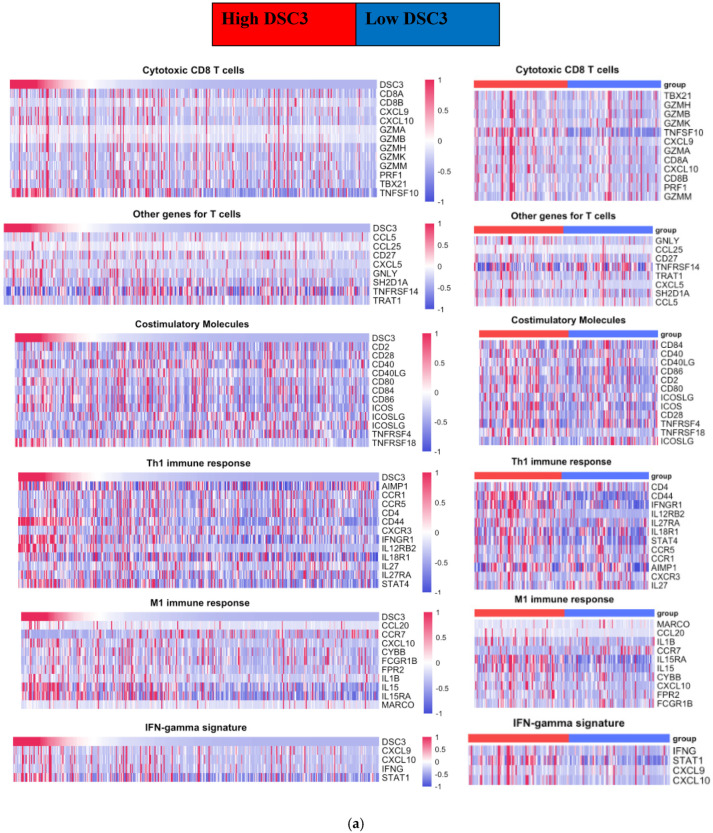

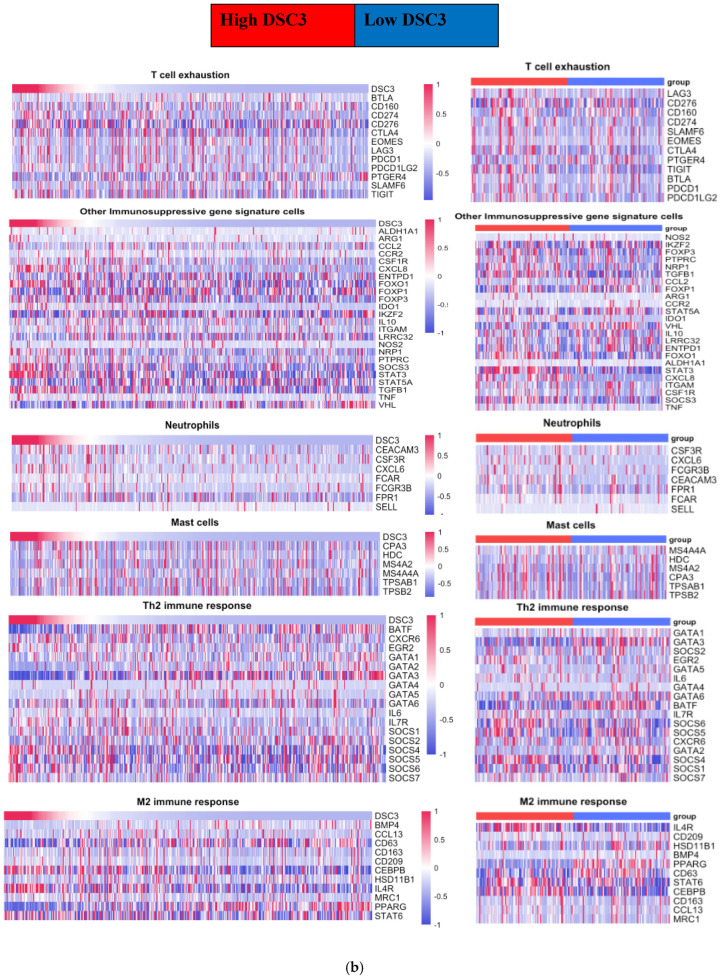
Response as per DSC3 expression level in MIBC. (**a**) Heat map for TCIG. Heat map showing the relationship between DSC3 gene expression and groups of tumor-controlling immune genes (TCIGs) in all samples of MIBC and also by high (upper quartile) and low (lower quartile) DSC3 expression. (**b**) Heat map for TPIG. Heat map showing the relationship between DSC3 gene expression and groups of tumor-promoting immune genes (TPIGs) in all samples of MIBC and also by high (upper quartile) and low (lower quartile) DSC3 expression.

To assess the immune profile of TIL linked with DSC3 expression, the relationship between 208 identified immune genes and DSC3 was analyzed. According to the function of immune genes, these genes were categorized into two primary groups (Table 6 and Table 7): tumor-controlling immune genes (TCIG, proinflammatory) and tumor-promoting immune genes (TPIG, immunosuppressive). Out of the 208 immune genes, only 37 (17.8%) were discovered to be associated with the DSC3 gene. Each of them exhibited a low to mild (≤0.6) correlation. Of the 37 immune genes, 16 belonged to the TCIG group and 21 to the TPIG group (Table 6 and Table 7 and Figure 2a,b).

Amongst correlated TCIG, two (TNFSF10 and CD44) had a positive correlation with DSC3 in both NMIBC and MIBC. The correlation coefficient was higher in MIBC (0.5) compared to NMIBC (0.3). All other correlated TCIG had a positive correlation in MIBC and a negative correlation in NMIBC.

Amongst correlated TPIG, all had a negative correlation with DSC3 in NMIBC. In MIBC, six had a positive correlation and seven had a negative correlation.

The number of TPIGs with a negative correlation with DSC3 was lower in MIBC (seven) compared to NMIBC (nine).

This is suggestive of increased immunosuppressive response associated with the progression of the disease.

Amongst genes representing T cell exhaustion, CTLA4 and TIGIT had a negative correlation with DSC3 expression in NMIBC. No correlation between other genes representing T cell exhaustion and DSC3 was observed in NMIBC. No correlation was observed between DSC3 and T cell exhaustion markers including inhibitory checkpoint genes (CTLA4 and TIGIT, CD244, EOMES, LAG3, PTGER4, CD274, PDCD1 (PD1), LG2, PDCD1, HAVCR2, BTLA, CD274 (PDL1), CD276, ENTPD1, and IDO1) in MIBC.

No correlation was observed between DSC3 and immune genes representing NK cells, B cells, innate lymphoid cells (ILC1, ILC2, and ILC3), inflammatory markers in NMIBC as well as MIBC.

Effect of DSC3 enrichment on tumor-infiltrating immune cell signature genes: (Table 6 and Table 7, Figure 2a,b and Supplementary Figure S3):

Since DSC3 protein expression is not seen in all samples of bladder cancer and DSC3 gene expression has a wider range (0-46509 RSEM), we evaluated the effect of DSC3 enrichment on tumor-infiltrating immune cell signature genes by analyzing TCIG and TPIG in the samples with DSC3 expression in the upper quartile (DSC3 enriched cohort) of all DSC3-expressing samples (whole cohort).

- NMIBC: Enrichment of DSC3 in NMIBC was associated with a decrease in the number of correlated TCIG and TPIG.

On DSC3 enrichment, two TCIG genes (TNFSF10, CD44) with a positive correlation across the whole cohort (Table 6 and Table 7) lost their correlation in this limited, high-DSC3 cohort. Similarly, of the six TCIG with a negative correlation across the whole cohort, only CTSW and IL1B retained the positive correlation in the smaller cohort with high expression of DSC3. Additionally, CCL15 (TPIG) was found to have a negative correlation on DSC3 enrichment only.

On DSC3 enrichment, only two of nine TPIG (MS4A2 and CCL13) with positive correlation retained their correlation. Additionally, IL4R was negatively correlated with DSC3 expression only in the cohort expressing high DSC3 levels.

The findings suggest that immune response to tumor cells is minimal in NMIBC expressing high-DSC3 genes.

b. MIBC: In contrast to NMIBC, we observed an increase in the number of TCIG having a positive correlation with DSC3 expression on DSC3-enrichment (9 to 14) and included CXCL10, ICOS, CCR7, IL1B, and CCL15 as additional DSC3 correlated TCIG. CCL15 was the only TCIG that converted from negative to positive correlation on DSC3 enrichment.

In contrast to TCIG, we observed a decrease in the no. of TPIGs with a positive correlation from six to five on DSC3 enrichment. CXCL6 lost its positive correlation on DSC3 enrichment. Of six negatively correlated TPIG, four had an increase in their correlation of BATF (0.3 to 0.6) and GATA2, GATA3, and PPARG from 0.4 to 0.6 on the enrichment of DSC3.

Heat map analysis on TCGA-BLCA patient data (Figure 2a,b) showed a visually noticeable correlation between DSC3 and TCIGs (including TNFSF10, CD44, IFNGR1, IL12RB2, STAT4, IL15, and STAT1) and TPIGs (including CXCL8, FOXP3, STAT3, VHL, BATF, GATA3, CEBPB, IL4R, CD63, and PPARG).

Additionally, genes with a moderate to high correlation (Spearman’s correlation ≥ 0.6) with DSC3 expression in the TCGA-BLCA dataset were identified on cBioPortal and analyzed for enrichment analysis (Supplementary Figure S3). Results suggest that the genes that are significantly correlated with DSC3 are involved in tissue barrier function and maintenance. Notably, the p53 signaling pathway was also enriched.

No correlation was seen between DSC3 and CD274 (PD-L1) or PDCD1 (PD-1) in any bladder cancer molecular subtype.

### 3.4. DSC3 Gene Expression and Pathological Classification

Among MIBC (TCGA dataset), DSC3 expression was higher in non-papillary MIBC (NPMIBC) compared to papillary MIBC (PMIBC) [mean RSEM 4187 NPMIBC vs. 1956 PMIBC; *p* = 0.001] (Table 1).

Among NPMIBC, DSC3 expression was significantly higher in pathologic squamous (pSquamous) than non-squamous [mean RSEM 11763 squamous NPMIBC vs. 2485 non-squamous NPMIBC; *p* < 0.0001].

pSquamous BC were predominantly NPMIBC (41 of 44; *p* = 0.0002. Chi-square statistic with Yates correction = 13.792).

**DSC3 and molecular classification of bladder cancer (** **Table 8** **):**

Because DSC3 expression has been previously identified in the basal/squamous (Ba/Sq) type of bladder cancer [3,4,5,6,7], we evaluated DSC3 expression level in basal squamous, neuronal, and luminal molecular subtypes of bladder cancer as defined in the TCGA dataset. We found that mean DSC3 values were less in luminal compared to basal, squamous, and neuronal bladder cancer subtypes.

To further elucidate the correlation between DSC3 and markers of bladder cancer molecular subtypes, we divided samples across molecular subtypes into DSC3 high (upper quartile (UQ)) and DSC3 low (lower quartile (LQ)) groups.

**Figure 3:**
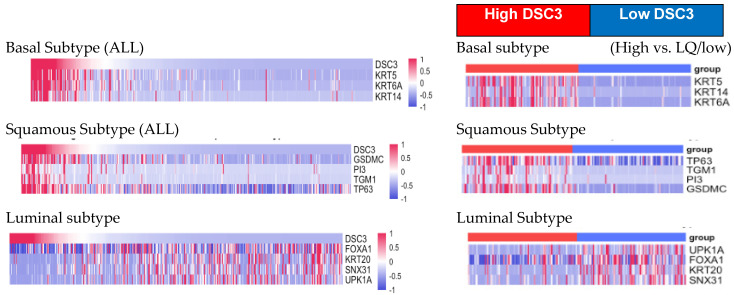
Heat map showing the relationship between DSC3 gene expression and genes for molecular subtype in all samples of MIBC and also by high (upper quartile) and low (lower quartile) DSC3 expression.

Samples in the UQ group (high DSC3 expression) had higher expression of markers for the basal (KRT5, KRT14, KRT6a) and squamous subtypes (TP63, TGM1, PI3, GSDMC) compared to the LQ group (lower DSC3 expression), which had very low expression of basal and squamous subtype markers (Figure 3). In contrast, markers for the luminal subtype were higher in the LQ group compared to the UQ group (Figure 3).

**DSC3 and P53:**

DSC3 is a p53-responsive gene, and so we evaluated the correlation of DSC3 expression with p53 status. We observed higher DSC3 gene expression levels in samples with mutant p53 compared to samples with wild-type p53 (Mean RSEM 4169.22 vs. 2517.96; *p* = 0.0285). Supporting this result, we also observed that genes involved in the p53 signaling pathway are over-represented and are highly correlated (spearman’s correlation = 1.0) with DSC3 (Supplementary Figure S3).

**DSC3 Expression and treatment outcome:**

**Figure 4:**
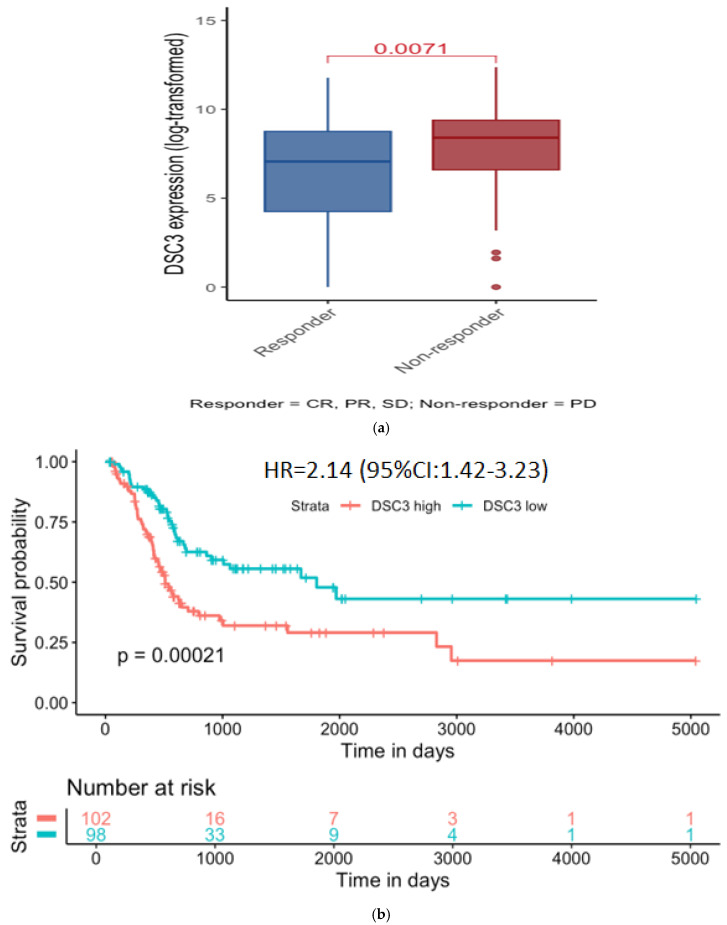
DSC3 expression and outcome. (**a**) DSC3 expression level in responders (CR+PR+SD) and non-responders. (**b**) Impact of DSC3 expression (high–upper quartile vs. low–lower quartile) on survival in MIBC patients (TCGA-BLCA).

Using TCGA-BRCA’s clinical data on primary treatment outcome, patient samples were grouped as responders (complete or partial response or stable disease) or non-responders (progressive disease). DSC3 gene expression levels were significantly (*p* = 0.0071, Wilcoxon test) higher in non-responders compared to responders (Figure 4a).

Because DSC3 was high in non-responders, we used a KM plotter to evaluate the impact of DSC3 expression on response to treatment. We found that higher DSC3 expression (UQ) was associated with poor survival while lower DSC3 expression (LQ) was associated with better outcomes (Figure 4b). The median overall survival was 60.13 months for the low DSC3 expression group (LQ) compared to only 17 months for the high-DSC3 group (UQ)(HR = 2.14 (95% CI 1.42–3.23); *p* = 0.00021).

**Figure 5:**
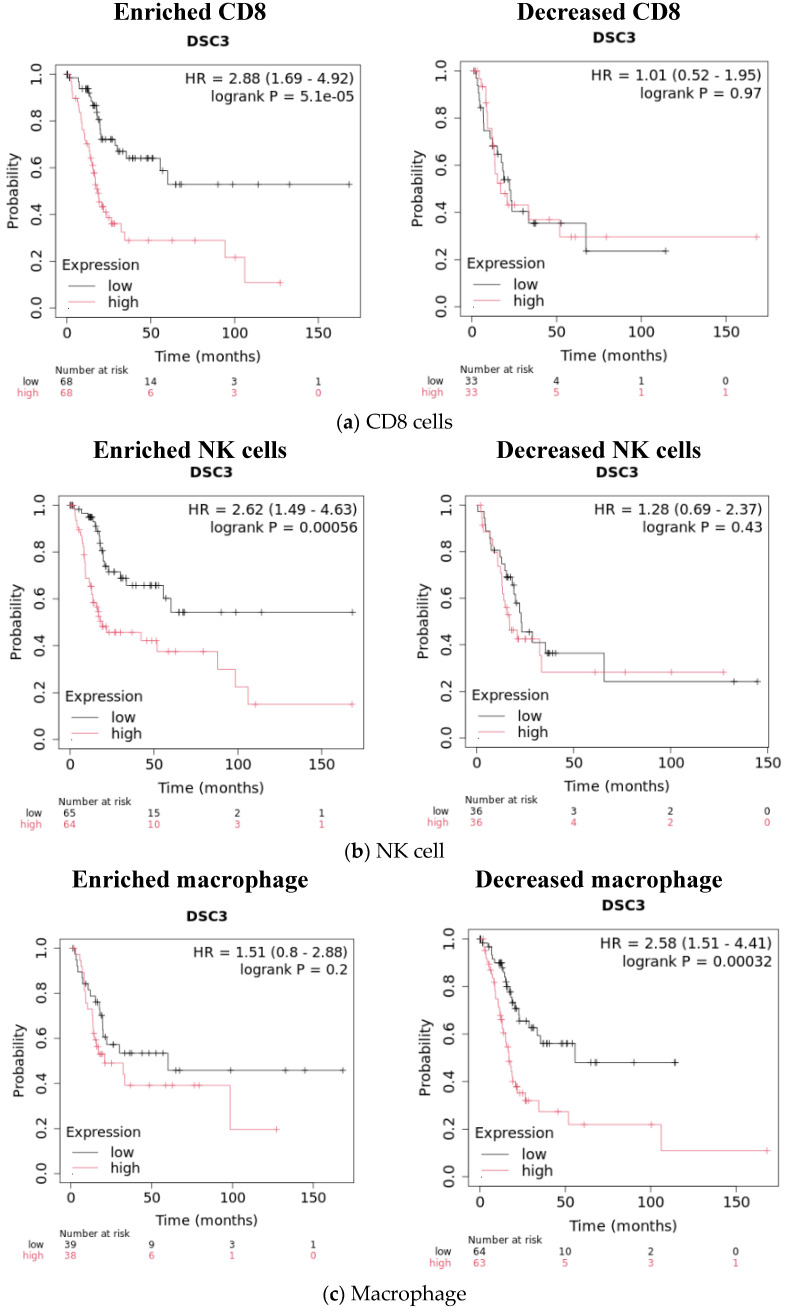
Impact of enrichment and decrease in immune cells in high and low DSC3-expressing MIBC on survival. (**a**) CD8 cells. (**b**) NK cells. (**c**) Macrophages.

The KM plotter is also useful in evaluating the effect (enrichment or decrease) of various immune parameters on survival (Figure 5 and Table 9). There was further improvement in survival in LQ compared to UQ on enrichment of CD8 (HR = 2.88; *p* = 0.000051) and NK cells (HR = 2.62; *p* = 0.00056). Further improvement in survival was also observed in the decrease in macrophages (HR = 2.62; *p* = 0.000320) and B cells (HR = 2.41; *p* = 0.0004). An inverse effect was seen in the decrease in regulatory T cells (HR = 1.71; *p* = 0.029).

## 4. Discussion

DSC3 gene expression has been described in MIBC. Its correlation with TIL and its impact on outcome is not described. In this study, we evaluated DSC3 protein and gene expression in NMIBC and MIBC. We also evaluated the relationship of DSC3 expression with tumor immune infiltrate and bladder cancer subtypes. For MIBC, we also evaluated its relationship with treatment outcome as well as survival using the TCGA dataset.

We observed a higher percentage of NIMBC tumors expressing DSC3 protein compared to MIBC (60% vs. 37.5%). In alignment with this observation of DSC3 protein expression, the mean DSC3 gene expression was also higher in NMIBC compared to MIBC. The decrease in DSC3 expression in MIBC seen in this study is consistent with a decrease in ΔNP63, which is a major isomer of TP63 and a key regulator of adhesion molecules that is closely related to DSC3 expression upon progression to MIBC [16,22]. Like DSC3, another adhesion molecule, E Cadherin, is also known to be decreased in MIBC [23].

Methylation of the DSC3 gene results in the loss of DSC3 protein [2]. An inverse correlation between DSC3 gene expression and its methylation (from TCGA) in this study suggests that DSC3 protein expression may be proportionate to DSC3 gene expression.

DSC3 gene expression has been described in Urobasal A, Urobasal B, squamous cell carcinoma, and Ba/Sq types of bladder cancer previously [5,7,8,9]. Ba/Sq bladder cancers are enriched in T cell exhaustion markers, cytotoxic T cells, NK cells, and M2 macrophages [5,7,8,24,25]. Moreover, increased PD-L1 tumor cell expression is almost exclusively seen in the basal subtype, but still only in 39% [25]. In this study, DSC3 gene expression was not associated with CD274 and PDCD1 in basal squamous subtype suggesting DSC3-expressing tumors as a distinct subset of basal/squamous bladder cancer.

The odds ratio for the presence of TIL in DSC3-expressing bladder cancer was 4.96-fold higher in MIBC compared to NMIBC (27.8 vs. 5.6). However, DSC3 protein expression was more frequent in NMIBC compared to MIBC (60.0% vs. 37.5%), with mean DSC3 gene expression 2.55-fold higher in NMIBC compared to MIBC (16.1 vs. 6.3 FPKM). One explanation of this negative correlation between DSC3 and TIL is the barrier function of DSC3, an adhesion molecule, toward immune infiltration as described in melanoma and ovarian cancer [26].

Unlike TIL, the presence of macrophage was not different in DSC3-expressing or non-expressing NMIBC and MIBC (Table 4 and Table 5). This may be attributed to the provision of innate immune protection as the bladder is an organ exposed to bacteria.

As per the immune editing hypothesis, the immune system attempts to control cancer growth by generating immune responses [27]. Cancer progression is associated with increased immune suppression. In this study, a positive correlation with TCIG and a negative correlation with TPIG on DSC3 enrichment (Table 6 and Table 7) suggests immune mechanisms may not play a significant role in DSC3-expressing bladder cancer.

Immunotherapy in the form of intravesical BCG to prevent the recurrence of NMIBC is the first approved immunotherapy. Subsequently, checkpoint inhibitors have been approved in the management of bladder cancer. All indicating a strong immune mechanism underlying the development and progression of bladder cancer. This has been corroborated in this study by the significant association of DSC3 protein expression with intratumoral TIL (odds ratio 15.467) compared to stromal TIL (odds ratio 5.663). However, the lack of correlation between DSC3 genes with T cell exhaustion markers including CD274 and PDCD1 improved negative correlation with TPIG, and a positive correlation with TCIG on DSC3 enrichment in MIBC suggests the possibility of other immune suppressive mechanisms or non-immune mechanisms responsible for tumor progression.

Immune checkpoint inhibitors, like anti-PD-1/PD-L1, have been approved for the treatment of urothelial cancers. PD-L1 expression is used as a biomarker for selection of patients [28]. Generally, the best responses to these therapies are seen when tumor antigens (neoantigens) experiencing TIL are exhausted [28]. In this study, intratumoral TILs did not express DSC3, suggesting TIL did not recognize DSC3 as a neoantigen [25]. Expression of DSC3 by normal cells also suggests that DSC3 is more likely to be a tumor-associated antigen rather than a neoantigen. In this study, we also observed the absence of a correlation between DSC3 gene expression and T cell exhaustion marker genes, including CD274 and PDCD1. The absence of DSC3 expression by TIL and lack of correlation between DSC3 and exhaustion marker suggest that DSC3-expressing BCs may not benefit from treatment with anti-PD-1/PD-L1 therapy. These DSC3-expressing BCs are likely to respond to CADI-05, as following administration of CADI-05, there is infiltration of activated CD8+T cells with a decrease in T cells expressing FoxP3, PD-1, and CTLA4 [11,12]. CADI-05 can also be combined with immune checkpoint inhibitors.

DSC3gene is a p53-dependent gene. Higher DSC3 expression in tumors with mutant p53 compared to wild-type p53, as seen in this study, is also described in squamous maxillary cancer and is contrary to its expression in lung cancer, colorectal, and unmethylated breast cancer [4,29,30,31,32].

Higher DSC3 gene expression was associated with poor survival in MIBC, despite a strong correlation (Spearman’s correlation = 1.0) with the p53 signaling pathway. This is contrary to its being a p53-responsive and tumor-suppressive gene [32,33]. Higher DSC3 expression in mutant p53 compared to wild-type p53 may be responsible for alteration in tumor-suppressive function. This relationship between DSC3 and survival is also described in melanoma and ovarian cancer [26].

DSC3 is closely associated with p63 and ΔNP63 expression [2,7]. Both are associated with poor survival in MIBC [7,34,35,36,37,38]. The poor outcome associated with DSC3 expression in MIBC, despite being a p53-responsive gene, is in line with poor survival described with p63 and ΔNP63 expression in MIBC.

The factors contributing to poor survival, in spite of being a p53-responsive gene having improved correlation with TCIG (+ve) and TPIG (−ve) on DSC3 enrichment, may include the following:

1. Its correlation with mutant p53 is seen in this study.
2. Positive correlation of DSC3 with stem cell marker CD44 and a negative correlation with GATA3 [39,40]. Gata3 is described to be associated with better outcomes in MIBC.

In conclusion, DSC3 expression is more frequent in NMIBC than in MIBC. DSC3 expression was associated with tumor-infiltrating lymphocytes in a stage-specific immunogenetic signature. However, DSC3 expression was not related to genes representing T cell exhaustion. Although the sample sizes are small in this study and immunohistochemistry was performed using commercially available non-validated reagents, our results highlight the importance of the DSC3 marker in understanding the nature of immunological differences in bladder cancer subtypes and cancer prognosis. The other limitations include reliance on the use of the TCGA dataset for bioinformatics analysis and lack of validation cohort. In spite of these limitations, the findings of the present study suggest that DSC3 can be a prognostic and predictive biomarker that needs independent validation.

## Figures and Tables

**Table 1:** DSC3 protein and gene expression across stages.

DSC3 Protein expression (immunohistochemistry)					
		Total N	Positive N (%)	Negative N (%)	Chi-square test (*p* value)
Stage	NMIBC	55	33 (60%)	22 (40%)	0.11
	MIBC	16	6 (37.5%)	10 (62.5%)	
DSC3 gene expression—In silico					
Database			Total N	Mean DSC3	*t* Test, 2 tail (*p* value)
ArrayExpress; E-MTAB-4321 (FKPM)	Stage	NMIBC	457	16.1	0.001
		MIBC	16	6.3	
TCGA; Cell 2017 & Cell 2017 supplement (RSEM)	Histology (Supplementary Table S3)	Papillary MIBC	132	1956.8	0.001
		Non-Papillary MIBC	270	4187.8	
		Squamous non-papillary MIBC	41	11,763	<0.0001
		Non-squamous non-papillary MIBC	229	2485	

**Table 2:** Correlation of DSC3 with TIL at protein levels.

A						
			**TIL**			
		**DSC3**	**Total (N)**	**Present(N)**	**Absent (N)**	**Odds Ratio** **(95% CI)** ***p***
		Positive	39	31(79.49%)	8 (20.51%)	5.663 (1.98 to 16.17) *p* = 0.001
		Negative	32	13(40.63%)	19(59.37%)	
Stage	NMIBC	Positive	33	25 (75.75%)	8 (24.24%)	3.75 (1.18 to 11.92) *p* = 0.0251
		Negative	22	10(45.45%)	12 (54.54%)	
	MIBC	Positive	6	6 (100%)	0	27.8571 (1.20 to 646.11) *p* = 0.0381
		Negative	10	3(30%)	7(70%)	

**Table 3:** Presence of stromal and intratumoral TIL with respect to DSC3 expression in bladder cancer samples.

	sTIL		
DSC3	Absent	Present	Odds Ratio (95% CI) *p*
Positive (N = 39)	8 (20.51%)	31 (79.49%)	5.663 (1.98 to 16.18) *p* = 0.001
Negative (N = 32)	19 (59.37%)	13 (40.63%)	
	**iTIL**		
DSC3	**Absent**	**Present**	Odds ratio (95% CI) *p*
Positive (N = 39)	15 (39.46%)	24 (61.54%)	15.467 (3.9997 to 59.8091) *p* = 0.000
**Negative** **(N = 32)**	29 (90.63%)	3 (9.37%)	

**Table 4:** Correlation of DSC3 with macrophage at protein levels.

			Macrophage			
		DSC3	Total (N)	Present (N)	Absent (N)	Odds Ratio (95% CI) *p*
Stage	NMIBC	Positive	33	32	1	0.4815 (0.01 to 12.36) *p* = 0.6589
		Negative	22	22	0	
	MIBC	Positive	6	6	0	0.6190 (0.01 to 35.18) *p* = 0.8160
		Negative	10	10	0	
		Negative	14	14	0	

**Table 5:** Presence of stromal and intratumoral macrophage with respect to DSC3 expression in bladder cancer samples.

	sMacrophage				
DSC3	Absent	Present	Odds Ratio (95% CI) *p*		
Negative (N = 32)	0	32	0.395	0.016 to 10.028	*p* = 0.573
Positive (N = 39)	1	38			
	**iMacrophage**				
**DSC3**	**Absent**	**Present**	**Odds Ratio** **(95% CI)** ***p***		
Negative (N = 32)	5	27	0.718	0.210 to 2.457	*p* = 0.597
Positive (N = 39)	8	31			

**Table 6:** Spearman correlation of immune function of TCIG with DSC3.

Subset of Immune Cells	Correlated Genes	NMIBC	UQ NMIBC	MIBC	UQ MIBC
Cytotoxic CD8 T cells	TNFSF10	0.3	0.2	0.5	0.4
	CXCL10	−0.3	−0.1	0.2	0.3
Other genes for T cells	CTSW	−0.3	−0.3	0.2	−0.2
Costimulatory Molecules	ICOS	−0.3	−0.1	0.2	0.3
	TNFRSF18	−0.1	0.0	0.3	0.4
Th1 immune response	CCR1	−0.3	−0.2	0.2	0.1
	CD44	0.3	0.1	0.5	0.6
	IFNGR1	0.1	0.0	0.3	0.4
	IL12RB2	−0.1	0.0	0.4	0.5
	STAT4	0	0.1	0.3	0.3
M1 immune response	CCL15	−0.2	−0.4	−0.4	0.4
	CXCL10	−0.3	−0.1	0.2	0.3
	CCL20	−0.2	−0.1	0.3	0.5
	CCR7	−0.3	−0.1	−0.2	0.3
	IL1B	−0.3	−0.3	0.1	0.3
	IL15	0	−0.1	0.3	0.4
IFN-gamma signature	CXCL10	−0.3	−0.1	0.2	0.3
	STAT1	−0.1	−0.2	0.3	0.3

(Blue color denots negative and green color denots positive correlations).

**Table 7:** Spearman correlation of immune function of TPIG with DSC3.

**Subset of Immune Cells**	**Gene**	**NMIBC**	**UQ NMIBC**	**MIBC**	**UQ MIBC**
T cell exhaustion	CTLA4	−0.3	−0.1	0.1	0.2
	TIGIT	−0.3	−0.1	0.2	0.2
Other Immunosuppressive gene signature cells	CXCL8	NA	NA	0.4	0.4
	FOXP3	−0.3	−0.1	0.1	0.1
	STAT3	0.1	0.1	0.3	0.4
	VHL	−0.3	−0.1	−0.3	−0.3
Neutrophil	CXCL6	−0.1	0.0	0.3	0.2
	SELL	−0.3	−0.1	−0.2	−0.2
	S100A12	−0.2	−0.1	0.3	0.4
Mast cells	CPA3	−0.3	−0.1	0.1	−0.1
	MS4A2	−0.3	−0.3	0.1	−0.1
Th2 immune response	BATF	−0.1	−0.1	−0.3	−0.6
	GATA2	0.1	−0.1	−0.4	−0.6
	GATA3	0.1	0.1	−0.4	−0.6
M2 immune response	BMP4	−0.2	0.0	−0.3	−0.3
	CCL13	−0.3	−0.3	0.1	0.0.
	CEBPB	0.1	−0.1	0.3	0.3
	IL4R	0.1	−0.3	0.3	0.3
	SIGLEC5	−0.3	−0.2	−0.2	−0.1
	CD63	0	−0.1	−0.3	−0.5
	PPARG	0	0.0	−0.4	−0.6

(Blue color denots negative and green color denots positive correlations).

**Table 8:** DSC3 gene expression and its correlation with the molecular subtype of MIBC.

		P MIBC	NPNPMIBCI	NMIBC	Squamous
**BASAL**	KRT5	**0.6**	**0.7**	**0.4**	0
	KRT6A	**0.5**	**0.7**	**0.4**	0.1
	KRT14	**0.3**	**0.6**	**−0.1**	**0.2**
	Average BASAL	**0.5**	**0.7**	**0.2**	**0.1**
**Squamous**	CD44	**0.3**	**0.5**	**0.3**	**0.2**
	GSDMC	**0.3**	**0.6**	**0**	**0.3**
	PI3	**0.1**	**0.6**	**−0.1**	**0.1**
	TGM1	**0.1**	**0.5**	**0**	**0.1**
	TP63	**0.2**	**0.6**	**0.4**	**0.3**
	Average Squamous	**0.2**	**0.6**	**0.12**	**0.2**
**Luminal**	PPARG	**−0.3**	**−0.5**	**0**	**−0.2**
	GATA3	**−0.2**	**−0.4**	**0.1**	**−0.1**
	KRT20	**−0.1**	**−0.3**	**−0.1**	0
	FOXA1	**−0.2**	**−0.2**	**0**	**−0.1**
	SNX31	**−0.1**	**−0.3**	**−0.1**	**−0.1**
	UPK1A	**−0.2**	**−0.3**	**−0.4**	**−0.2**
	UPK2	**−0.3**	**−0.4**	**−0.4**	**−0.2**
	Average Luminal	−0.2	−0.3	−0.1	−0.1

(Blue color denots negative and green color denots positive correlations).

**Table 9:** Effect of various immune parameters on survival of MIBC as per DSC3 expression level high vs. low (UQ vs. LQ).

	HR	Log Rank *p* Value	N = Low	N = High
UQ vs. LQ DSC3	2.14 (1.42–3.23)	0.0002	101	101
Effect of enrichment or decrease on survival in UQ and LQ DSC3				
CD8 enrichment	2.88 (1.69–4.92)	0.00005	68	68
NK cell enrichment	2.62 (1.49–4.63)	0.00056	65	64
Macrophage decreased	2.58 (1.51–4.41)	0.00032	64	63
B cell decreased	2.41 (1.46–3.99)	0.0004	63	62
Regulatory T cell decreased	1.71 (1.05–2.79)	0.029	71	71

## Data Availability

All data has been included.

## Supplementary Materials

The following supporting information can be downloaded at: https://www.mdpi.com/article/10.3390/diseases13050131/s1, Table S1: Details of Institutional Ethics Committees and approval for clinical study. Table S2: List of genes used for analysis of immune signature and subset of immune cells. Table S3: Histopathological evaluation of Bladder cancer samples. Figure S1: DSC3 expression in bladder cancer. Figure S2: heat map showing the relationship between DSC3 gene expression and other immune genes in all samples of MIBC and also by high (upper quartile) and low (lower quartile) DSC3 expression. Figure S3: Enrichment analysis with DSC3Gs (Spearman’s correlation ≥ 0.6) in bladder cancer.

## Abbreviations

DSC3:	Desmocollin-3
BC:	Bladder cancer
TIL:	Tumor-infiltrating lymphocytes
NMIBC:	Non-muscle-invasive bladder cancer
MIBC:	Muscle-invasive bladder cancer
Ba/Sq:	Basal/squamous
TII:	Tumor immune infiltrate
EDTA:	Ethylenediamine tetraacetic acid
pH:	Potential of hydrogen
HRP:	Horseradish peroxidase
DAB:	3,3′-Diaminobenzidine
H&E:	Haematoxylin and eosin
RSEM:	RNAseq by expectation-maximization
mRNA:	Messenger RNA
FPKM:	Fragments per kilobase of transcript per million mapped reads
HRNMIBC:	High-risk NMIBC
CI:	Confidence interval
TCGA:	The Cancer Genome Atlas
BLCA:	Bladder urothelial carcinoma
OS:	Overall survival
HR:	Hazard ratio
pSquamous:	Pathologic squamous
DSC3G:	DSC3 correlated genes

## References

[1] Gumbiner B.M. (1996). Cell Adhesion: The Molecular Basis of Tissue Architecture and Morphogenesis. Cell.

[2] Shukla C., Jain N.K., Khamar B., Atta-ur-Rahman (2021). Chapter 4- Desmocollin-3 and Cancer. Frontiers in Clinical Drug Research—Anti-Cancer Agents.

[3] Mauro T. (2014). Endoplasmic reticulum calcium, stress, and cell-to-cell adhesion. J. Investig. Dermatol..

[4] Kudo I., Esumi M., Yoshiaki K., Tohru F., Oshima T. (2017). Particular gene upregulation and p53 heterogeneous expression in TP53-mutated maxillary carcinoma. Oncol. Lett..

[5] Mo Q., Li R., Adeegbe D.O., Peng G., Chan K.S. (2020). Integrative multi-omics analysis of muscle-invasive bladder cancer identifies prognostic biomarkers for frontline chemotherapy and immunotherapy. Commun. Biol..

[6] Robertson A.G., Kim J., Al-Ahmadie H., Bellmunt J., Guo G., Cherniack A.D., Hinoue T., Laird P.W., Hoadley K.A., Akbani R. (2017). Comprehensive Molecular Characterization of Muscle-Invasive Bladder Cancer. Cell.

[7] Kim B., Jang I., Kim K., Jung M., Lee C., Park J.H., Kim Y.A., Moon K.C. (2021). Comprehensive Gene Expression Analyses of Immunohistochemically Defined Subgroups of Muscle-Invasive Urinary Bladder Urothelial Carcinoma. Int. J. Mol. Sci..

[8] Tan T.Z., Rouanne M., Tan K.T., Huang R.Y.J., Thiery J.P. (2019). Molecular Subtypes of Urothelial Bladder Cancer: Results from a Meta-cohort Analysis of 2411 Tumors. Eur. Urol..

[9] Inamura K. (2018). Bladder Cancer: New Insights into Its Molecular Pathology. Cancers.

[10] O’donnell M.A., Singh S., Sood R., Amlani J., Krishnamoorthy H., Shukla K., Mohanty N., Bhatia S., Chakraborty B., Desai N. (2019). A Clinical Trial of the Intradermal TLR2 Agonist CADI-05 for BCG Recurrent and Unresponsive Non-Muscle Invasive Bladder Cancer. Bladder Cancer.

[11] Ahmad F., Mani J., Kumar P., Haridas S., Upadhyay P., Bhaskar S. (2011). Activation of anti-tumor immune response and reduction of regulatory T cells with Mycobacterium indicus pranii (MIP) therapy in tumor bearing mice. PLoS ONE.

[12] Roy G., Chakraborty A., Swami B., Pal L., Ahuja C., Basak S., Bhaskar S. (2023). Type 1 interferon mediated signaling is indispensable for eliciting anti-tumor responses by Mycobacterium indicus pranii. Front. Immunol..

[13] Rakshit S., Ponnusamy M., Papanna S., Saha B., Ahmed A., Nandi D. (2012). Immunotherapeutic efficacy of Mycobacterium indicus pranii in eliciting anti-tumor T cell responses: Critical roles of IFNγ. Int. J. Cancer.

[14] Hendry S., Salgado R., Gevaert T., Russell P.A., John T., Thapa B., Christie M., van de Vijver K., Estrada M.V., Gonzalez-Ericsson P.I. (2017). Assessing Tumor-infiltrating Lymphocytes in Solid Tumors: A Practical Review for Pathologists and Proposal for a Standardized Method From the International Immunooncology Biomarkers Working Group: Part 1: Assessing the Host Immune Response, TILs in Invasive Breast Carcinoma and Ductal Carcinoma In Situ, Metastatic Tumor Deposits and Areas for Further Research. Adv. Anat. Pathol..

[15] Chiu Y.J., Hsieh Y.H., Huang Y.H. (2019). Improved cell composition deconvolution method of bulk gene expression profiles to quantify subsets of immune cells. BMC Med. Genom..

[16] Gao J., Aksoy B.A., Dogrusoz U., Dresdner G., Gross B.E., Sumer S.O., Sun Y., Jacobsen A., Sinha R., Larsson E. (2013). Integrative Analysis of Complex Cancer Genomics and Clinical Profiles Using the cBioPortal. Sci. Signal..

[17] Antonio C., Tiago C.S., Catharina O., Luciano G., Claudia C., Davide G., Thais S.S., Tathiane M.M., Stefano M.P., Isabella C. (2016). TCGAbiolinks: An R/Bioconductor package for integrative analysis of TCGA data. Nucleic Acids Res..

[18] Silva T.C., Colaprico A., Olsen C., D’Angelo F., Bontempi G., Ceccarelli M., Noushmehr H. (2016). TCGA Workflow: Analyze cancer genomics and epigenomics data using Bioconductor packages. F1000Research.

[19] Mounir M., Lucchetta M., Silva T.C., Olsen C., Bontempi G., Chen X., Noushmehr H., Colaprico A., Papaleo E. (2019). New functionalities in the TCGAbiolinks package for the study and integration of cancer data from GDC and GTEx. PLoS Comput. Biol..

[20] Alboukadel K., Marcin K., Biecek P. (2020). Drawing Survival Curves Using ggplot2 [R Package Survminer Version 0.4.8]. https://api.semanticscholar.org/CorpusID:226195259.

[21] Jeremy S. Social Science Statistics (Updated 2018). https://www.socscistatistics.com/tests/spearman/default2.aspx.

[22] Hurst C.D., Cheng G., Platt F.M., Mauro A.A.C., Nour-al-dain S.M., Pontus E., Emma V.I.B., Olivia A., Andrew R.J.L., Sia V.L. (2021). Stage-stratified molecular profiling of non-muscle-invasive bladder cancer enhances biological, clinical, and therapeutic insight. Cell Rep. Med..

[23] Xie Y., Li P., Gao Y., Gu L., Chen L., Fan Y., Zhang F., Zhang X. (2017). Reduced E-cadherin expression is correlated with poor prognosis in patients with bladder cancer: A systematic review and meta-analysis. Oncotarget.

[24] Kamoun A., de Reyniès A., Allory Y., Sjödahl G., Robertson A.G., Seiler R., Hoadley K.A., Groeneveld C.S., Al-Ahmadie H., Choi W. (2020). A Consensus Molecular Classification of Muscle-invasive Bladder. Eur. Urol..

[25] Rosenberg J.E., Hoffman-Censits J., Powles T., van der Heijden M.S., Balar A.V., Necchi A., Dawson N., O’Donnell P.H., Balmanoukian A., Loriot Y. (2016). Atezolizumab in patients with locally advanced and metastatic urothelial carcinoma who have progressed following treatment with platinum-based chemotherapy: A single-arm, multicentre, phase 2 trial. Lancet.

[26] Elise P.S., Davide B., Ileana S.M., Donna H.D., Sofia M.S., Joel P., Joseph M.O., George C., Ena W., Thomas F.G. (2016). Human melanomas and ovarian cancers overexpressing mechanical barrier molecule genes lack immune signatures and have increased patient mortality risk. OncoImmunology.

[27] O’Donnell J.S., Teng M.W.L., Smyth M.J. (2018). Cancer immunoediting and resistance to T cell-based immunotherapy. Nat. Rev. Clin. Oncol..

[28] Karunamurthy A., Davar D. (2025). There and back again: PD-L1 Positivity as a Biomarker for Immune Checkpoint Blockade in Urothelial Carcinoma. Cancer Immunol. Res..

[29] Cui T., Chen Y., Yang L., Knösel T., Zöller K., Huber O., Petersen I. (2011). DSC3 expression is regulated by p53, and methylation of DSC3 DNA is a prognostic marker in human colorectal cancer. Br. J. Cancer.

[30] Cui T., Yang L., Ma Y., Petersen I., Yuan C. (2019). Desmocollin 3 has a tumor suppressive activity through inhibition of AKT pathway in colorectal cancer. Exp. Cell Res..

[31] Cui T., Chen Y., Yang L., Knösel T., Huber O., Pacyna-Gengelbach M., Petersen I. (2012). The p53 target gene desmocollin 3 acts as a novel tumor suppressor through inhibiting EGFR/ERK pathway in human lung cancer. Carcinogenesis.

[32] Oshiro M.M., Watts G.S., Wozniak R.J., Junk D.J., Munoz-Rodriguez J.L., Domann F.E., Futscher B.W. (2003). Mutant p53 and aberrant cytosine methylation cooperate to silence gene expression. Oncogene.

[33] Riker A.I., Enkemann S.A., Fodstad O., Liu S., Ren S., Morris C., Xi Y., Howell P., Metge B., Samant R.S. (2008). The gene expression profiles of primary and metastatic melanoma yields a transition point of tumor progression and metastasis. BMC Med. Genom..

[34] Choi W., Shah J.B., Tran M., Svatek R., Marquis L., Lee I.L., Yu D., Adam L., Wen S., Shen Y. (2012). p63 expression defines a lethal subset of muscle-invasive bladder cancers. PLoS ONE.

[35] Yang L., Li A., Liu F., Zhao Q., Ji S., Zhu W., Yu W., Zhang R., Liu Y., Li W. (2021). Immune Profiling Reveals Molecular Classification and Characteristic in Urothelial Bladder Cancer. Front. Cell Dev. Biol..

[36] Eckstein M., Strissel P., Strick R., Weyerer V., Wirtz R., Pfannstiel C., Wullweber A., Lange F., Erben P., Stoehr R. (2020). Cytotoxic T-cell-related gene expression signature predicts improved survival in muscle-invasive urothelial bladder cancer patients after radical cystectomy and adjuvant chemotherapy. J. Immunother. Cancer.

[37] McConkey D.J., Choi W., Shen Y., Lee I.L., Porten S., Matin S.F., Kamat A.M., Corn P., Millikan R.E., Dinney C. (2016). A Prognostic Gene Expression Signature in the Molecular Classification of Chemotherapy-naïve Urothelial Cancer is Predictive of Clinical Outcomes from Neoadjuvant Chemotherapy: A Phase 2 Trial of Dose-dense Methotrexate, Vinblastine, Doxorubicin, and Cisplatin with Bevacizumab in Urothelial Cancer. Eur. Urol..

[38] Wang L., Zhou M., Feng C., Gao P., Ding G., Zhou Z., Jiang H., Wu Z., Ding Q. (2016). Prognostic value of Ki67 and p63 expressions in bladder cancer patients who underwent radical cystectomy. Int. Urol. Nephrol..

[39] Wang C.C., Tsai Y.C., Jeng Y.M. (2019). Biological significance of GATA3, cytokeratin 20, cytokeratin 5/6 and p53 expression in muscle-invasive bladder cancer. PLoS ONE.

[40] Choi W., Porten S., Kim S., Willis D., Plimack E.R., Hoffman-Censits J., Roth B., Cheng T., Tran M., Lee I.L. (2014). Identification of distinct basal and luminal subtypes of muscle-invasive bladder cancer with different sensitivities to frontline chemotherapy. Cancer Cell.

