# Supplementary material for: Desmocollin-3 and Bladder Cancer
Source: Diseases. 2025 Apr 23;13(5):131. doi: 10.3390/diseases13050131 (PMC12109863; doi:10.3390/diseases13050131)
Supplement: Supplementary file 1 [file diseases-13-00131-s001.zip › diseases-3483327-supplementary.pdf]

Supplementary Table 2: List of genes used for analysis of immune signature and subset of immune cells

**B cells**									
BLK	CD19	FAM30A	FCRL2	MS4A1	NFAM1	PNOC	SPIB	TCL1A	TNFRSF17
**Costimulatory Molecules**									
CD2	CD28	CD40	CD40LG	CD80	CD84	CD86	ICOS	ICOSLG	TNFRSF18
TNFRSF4									
**Cytotoxic CD8 T cells**									
CD8A	CD8B	CXCL10	CXCL9	GZMA	GZMB	GZMH	GZMK	GZMM	PRF1
TBX21	TNFSF10								
**IFN-gamma signature**									
CXCL10	CXCL9	STAT1	IFNG						
**ILC1 related genes**									
CD69	ITGA1	ITGAE	NCAM1	ZNF683					
**ILC2 related genes**									
AREG	HNF1A	IL13	IL2RA	IL33	ISG20	KLRG1	PTGDR2	RORA	
**ILC3 related genes**									
AHR	CCR6	IL17A	IL26	KIT	NCR2	RORC			
**Immune associated**									
CDKN2A	DNMT3B	FGFR3	TP53	TP63	TP73				
**Inflammatory**									
DDX58	IL10RB	IL11RA	IL12A	IL12B	IL13RA1	IL13RA2	KLRB1	KLRD1	KLRK1
MKI67	MX1	NKG7	SPP1	TOLLIP					
**M1 immune response**									
CCL15	CCL20	CCR7	CXCL10	CYBB	FCGR1B	FPR2	IL15	IL15RA	IL1B
MARCO									
**M2 immune response**									
BMP4	CCL13	CD163	CD209	CD63	CD68	CEBPB	HSD11B1	IL4R	MRC1
PPARG	SIGLEC5	STAT6							
**Mast cells**									
CPA3	HDC	MS4A2	MS4A4A	TPSAB1	TPSB2				
**Neutrophils**									
CEACAM3	CSF3R	CXCL6	FCAR	FCGR3B	FPR1	S100A12	SELL	CXCL1	
**NK cells**									
CD244	IL21R	KIR2DL3	KIR3DL1	KIR3DL2	NCR1	XCL2			
**Other genes for T cells**									
CCL19	CCL21	CCL25	CCL5	CD27	CD3D	CD3E	CD3G	CTSW	CXCL3
CXCL5	GNLY	SH2D1A	TNFRSF14	TRAT1					
**Other Immunosuppressive gene signature cells**									
ALDH1A1	ARG1	CCL2	CCR2	CSF1R	CSF2	CXCL8	ENTPD1	FOXO1	FOXP1
FOXP3	IDO1	IKZF2	IL10	IL4	IL5	ITGAM	IZUMO1R	LRRC32	NOS2
NRP1	PTPRC	SOCS3	STAT3	STAT5A	TGFB1	TNF	VHL		

**T cell exhaustion**									
BTLA	CD160	CD274	CD276	CTLA4	EOMES	LAG3	PDCD1	PDCD1LG2	PTGER4
SLAMF6	TIGIT	VSIR							
**Th1 immune response**									
AIMP1	CCR1	CCR5	CD4	CD44	CXCR3	IFNGR1	IL12RB2	IL18R1	IL27
IL27RA	STAT4								
**Th2 immune response**									
BATF	CXCR6	EGR2	GATA1	GATA2	GATA3	GATA4	GATA5	GATA6	IL6
IL7R	SOCS1	SOCS2	SOCS4	SOCS5	SOCS6	SOCS7			
**List of genes correlated with DSC3 for basal subtype of cancer**									
KRT5	KRT6A	KRT14							
**List of genes correlated with DSC3 for squamous subtype of cancer**									
GSDMC	PI3	TGM1	TP63						
**List of genes correlated with DSC3 for luminal subtype of cancer**									
KRT20	FOXA1	SNX31	UPK1A	UPK2					

Supplementary Table 3: Histopathological evaluation of Bladder cancer samples

Sr. No.	Patient ID/Slide ID	Stage	DSC-3 staining	TIL-Stromal	TIL-Intra-tumoral	Macrophage-Stromal	Macrophage-Intra-tumoral
1	916/12(1136)	MIBC	- ve	-ve	-ve	+Ve	+Ve
2	917/13(209)	MIBC	+Ve	+ve	+Ve	+Ve	+Ve
3	918/13(39)	MIBC	+Ve	+ve	+Ve	+Ve	+Ve
4	920/13(87)	MIBC	- ve	-ve	-ve	+Ve	+Ve
5	921/13(102)	MIBC	+Ve	+ve	-ve	+Ve	-ve
6	927/13(1943)	MIBC	+Ve	+ve	+Ve	+Ve	+Ve
7	928/13(2225)	MIBC	+Ve	+ve	+Ve	+Ve	+Ve
8	929/13(2707/12)	MIBC	+Ve	+ve	-ve	+Ve	+Ve
9	1345 (4263)	MIBC	- ve	+ve	-ve	+Ve	+Ve
10	1346 (2757)	MIBC	- ve	+ve	-ve	+Ve	+Ve
11	1348 (2577)	MIBC	- ve	-ve	-ve	+Ve	-ve
12	1351 (4518)	MIBC	- ve	-ve	-ve	+Ve	-ve
13	2912/12 (598/13)(1365)(598)	MIBC	- ve	-ve	-ve	+Ve	+Ve
14	2914/13 (946/13)(1366)	MIBC	- ve	-ve	-ve	+Ve	+Ve
15	2917/13 (1410/13)(1367)	MIBC	- ve	+ve	-ve	+Ve	+Ve
16	2917/13 (1412/13)(1363)	MIBC	- ve	-ve	-ve	+Ve	+Ve
17	922/13(214)	T1	+Ve	+ve	+Ve	+Ve	+Ve
18	925/13(1426/12)	T1	+Ve	-ve	-ve	+Ve	+Ve
19	926/13(1884)	T1	+Ve	-ve	-ve	+Ve	+Ve
20	1347 (2854)	T1	+Ve	+ve	+Ve	+Ve	+Ve
21	2918/13 (2219/13)(1368)	T1	- ve	-ve	-ve	+Ve	+Ve
22	2043/11	T1	- ve	-ve	-ve	+Ve	-ve
23	2472/11A (08-011)	T1	- ve	+ve	-ve	+Ve	+Ve
24	3387/11A(08-019)	T1	+Ve	+ve	+Ve	+Ve	+Ve
25	8486/12 low A (03-022)	T1	+Ve	+ve	+Ve	+Ve	+Ve
26	0A-2 (665/17)(16002)	T1	+Ve	+ve	+Ve	+Ve	+Ve
27	3001(651/17 MCH)	T1	+Ve	+ve	+Ve	+Ve	-ve
28	438/10 MLH (657/17)(10002)	T1	+Ve	+ve	-ve	+Ve	+Ve
29	661/17(1401)(12001)	T1	- ve	-ve	-ve	+Ve	+Ve
30	669/17 0A3(16003)	T1	+Ve	+ve	+Ve	+Ve	-ve
31	15003MLh (666/17)	T1	+Ve	+ve	-ve	+Ve	+Ve
32	668/17 MLH (15002)	T1	+Ve	+ve	+Ve	+Ve	+Ve
33	807 MCH (9002)	T1	- ve	+ve	+Ve	+Ve	+Ve
34	919/13(50)	Ta	+Ve	-ve	-ve	+Ve	-ve
35	923/13(422)	Ta	+Ve	+ve	+Ve	+Ve	+Ve
36	924/13(1287/12)	Ta	+Ve	+ve	+Ve	+Ve	+Ve
37	1349 (3624)	Ta	- ve	+ve	+Ve	+Ve	+Ve

38	1350 (1302)	Ta	- ve	-ve	-ve	+Ve	+Ve
39	1352 (3686)	Ta	- ve	-ve	-ve	+Ve	+Ve
40	2913/13 (867/13)(1361)	Ta	- ve	-ve	-ve	+Ve	+Ve
41	2915/13 (1170/13)(1362)	Ta	- ve	+ve	-ve	+Ve	+Ve
42	2916/13 (2160/13)(1364)	Ta	- ve	-ve	-ve	+Ve	-ve
43	755 MCH (broken slide)	Ta	- ve	+ve	-ve	+Ve	+Ve
44	788 MCH (789)(09-007)	Ta	- ve	-ve	-ve	+Ve	+Ve
45	07 MCH	Ta	- ve	+ve	-ve	+Ve	+Ve
46	421/11B (08-007)	Ta	+Ve	-ve	-ve	+Ve	-ve
47	2977/10B (08-008)	Ta	- ve	+ve	-ve	+Ve	+Ve
48	2588-11A(08-012)	Ta	- ve	-ve	-ve	+Ve	+Ve
49	3830/11(08-013)	Ta	+Ve	-ve	-ve	+Ve	+Ve
50	2924/11A(08-015)	Ta	+Ve	+ve	+Ve	+Ve	+Ve
51	3470-11B(08-018)	Ta	+Ve	+ve	+Ve	+Ve	+Ve
52	3512(03-003)	Ta	- ve	+ve	-ve	+Ve	+Ve
53	3514(03-007)	Ta	+Ve	+ve	+Ve	+Ve	+Ve
54	3561(03-008)	Ta	- ve	-ve	-ve	+Ve	+Ve
55	4160(03-010)	Ta	+Ve	-ve	-ve	-ve	-ve
56	2129(03-011)	Ta	+Ve	-ve	-ve	+Ve	+Ve
57	618(03-012)	Ta	+Ve	+ve	+Ve	+Ve	+Ve
58	1724A(03-015)	Ta	+Ve	+ve	+Ve	+Ve	+Ve
59	5407/12(03-016)	Ta	+Ve	+ve	-ve	+Ve	+Ve
60	9071/12(03-020)	Ta	+Ve	+ve	+Ve	+Ve	+Ve
61	10718/18 low B (03-021)	Ta	+Ve	+ve	+Ve	+Ve	+Ve
62	1533/12(03-023)	Ta	+Ve	+ve	+Ve	+Ve	+Ve
63	1411(13003)	Ta	+Ve	+ve	-ve	+Ve	+Ve
64	652/17(9001)	Ta	- ve	-ve	-ve	+Ve	+Ve
65	658/17(10003 A)	Ta	+Ve	-ve	-ve	+Ve	-ve
66	1811(13002)	Ta	- ve	+ve	+Ve	+Ve	+Ve
67	655/17(1000/A)	Ta	- ve	-ve	-ve	+Ve	-ve
68	1712(13001)	Ta	+Ve	+ve	+Ve	+Ve	+Ve
69	660/17(10004)	Ta	+Ve	+ve	-ve	+Ve	-ve
70	3592/11C(08-020)	Ta	- ve	+ve	-ve	+Ve	+Ve
71	667/17 MLH (16001)(0A-1)	Ta	+Ve	+ve	+Ve	+Ve	+Ve

Supplementary Table -1 Details of Institutional Ethics committes and approval for clinical study.

A. Ethics committee Name	B. Approval code	C. Approval date
Muljibhai Patel Society for research in Nephro-Urology Ethics committee	-	07.06.2008
Office of Ethics Committee – V.M. Medical College & Safdarjang Hospital	21-09-EC(2/12)	25.11.2009
Ethics committee, S.P. Medical college & A.G. Hospital, Bikaner	-	20.11.2008
Ethics committee for research on human research – Seth G.S. Medical College & K.E.M Hospital	EC/OUT/1709/08	22.12.2008
Choithram hospital & Research Centre	CHR/2507	08.08.2008
IEC-Christian Medical College	-	25.08.2008
IEC-Cancer Hospital & Research hospital, Gwalior	-	25.05.2009
Independent Ethics Committee, Ahmedabad	-	23.08.2009
IEC-Institute of Post Graduate Medical Education & Research, Kolkata	Inst./IEC/691	11.11.2009
Independent Ethics Committee, Ahmedabad	-	04.01.2010
IEC- Postgraduate Institute of Medical Education & Research, Chandigarh	09/5257	08.10.2009
IEC-Indira Gandhi Hospital, Shimla	09-11758	10.12.1009
IEC- Postgraduate Institute of Medical Education & Research, Dr. Ram Manohar Lohia Hospital, New Delhi	18-61/06-RMLH(HA-I)/IEC/Vol.II/215	01.12.2009
Ethics committee-Kasturba Hospital, Manipal	IEC 198/2009	13.01.2010
IEC-NRR Hospital, Bangalore	-	23.08.2010

Supplementary Figure 1: DSC3 expression in bladder cancer

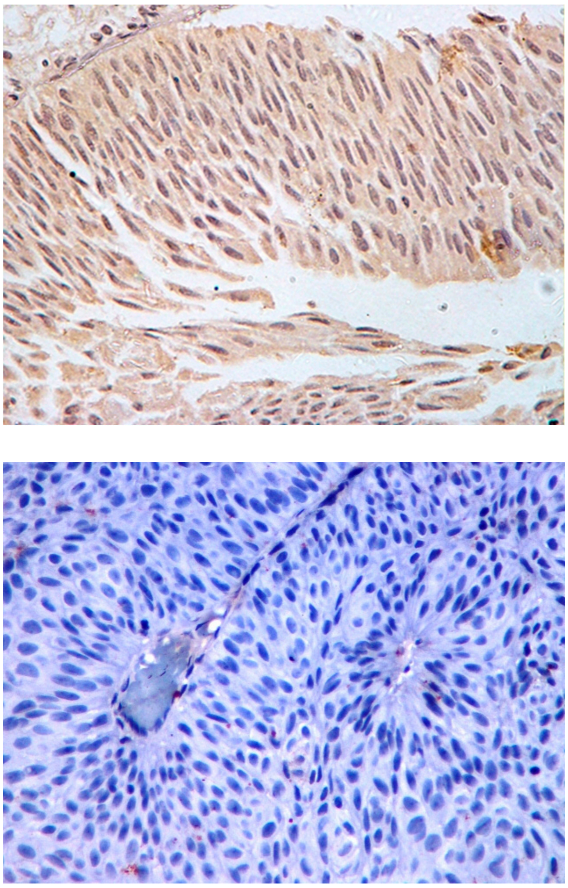

DSC3 expressing bladder cancer

DSC3 negative bladder cancer

Supplementary Figure 2. heat map showing relationship between DSC3 gene expression and other immune genes in all samples of MIBC and also by high (upper quartile) and low (lower quartile) DSC3 expression

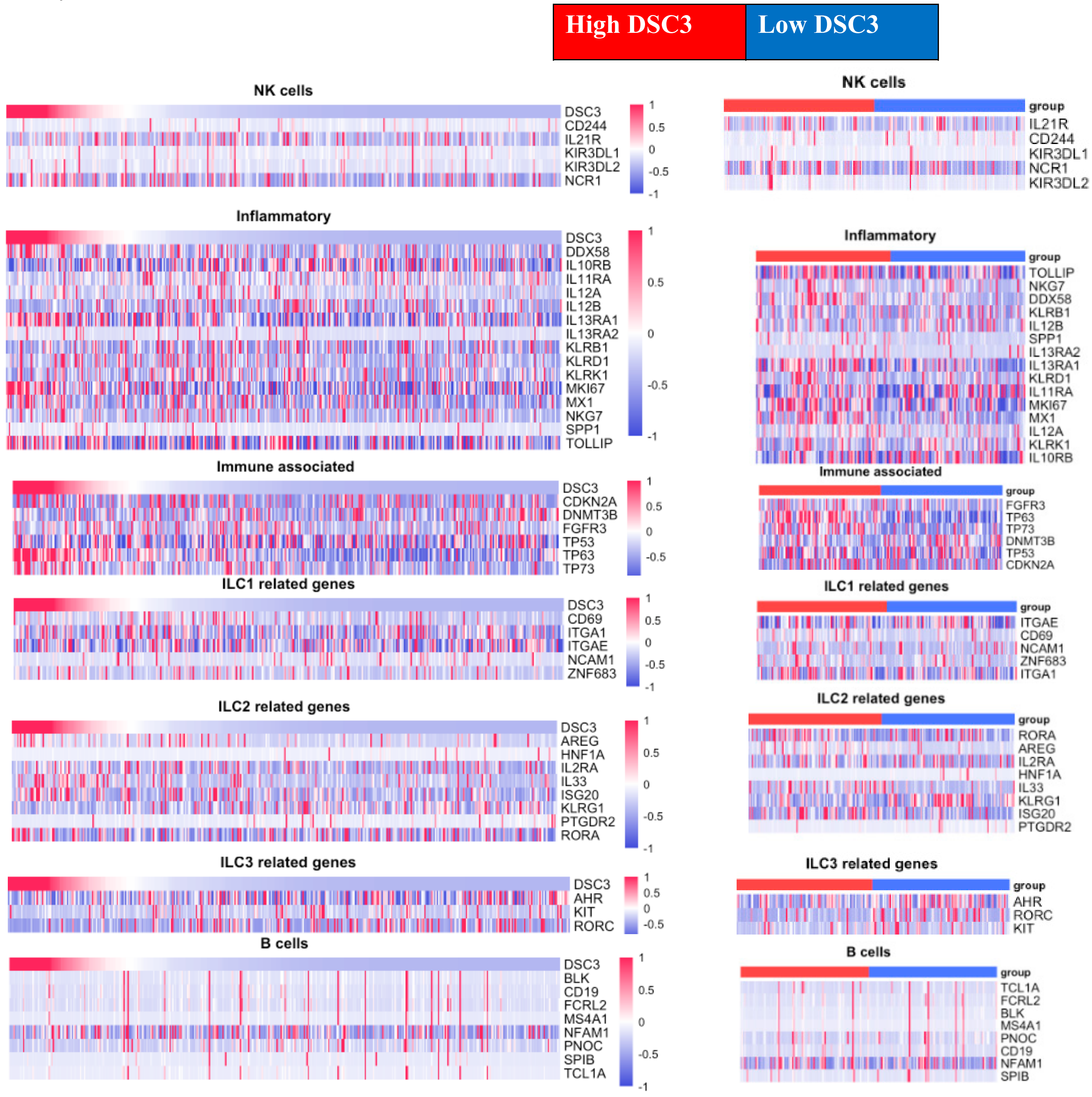

Supplementary Figure 3: Enrichment analysis with DSC3Gs (|Spearman's correlation| ≥ 0.6) in bladder cancer

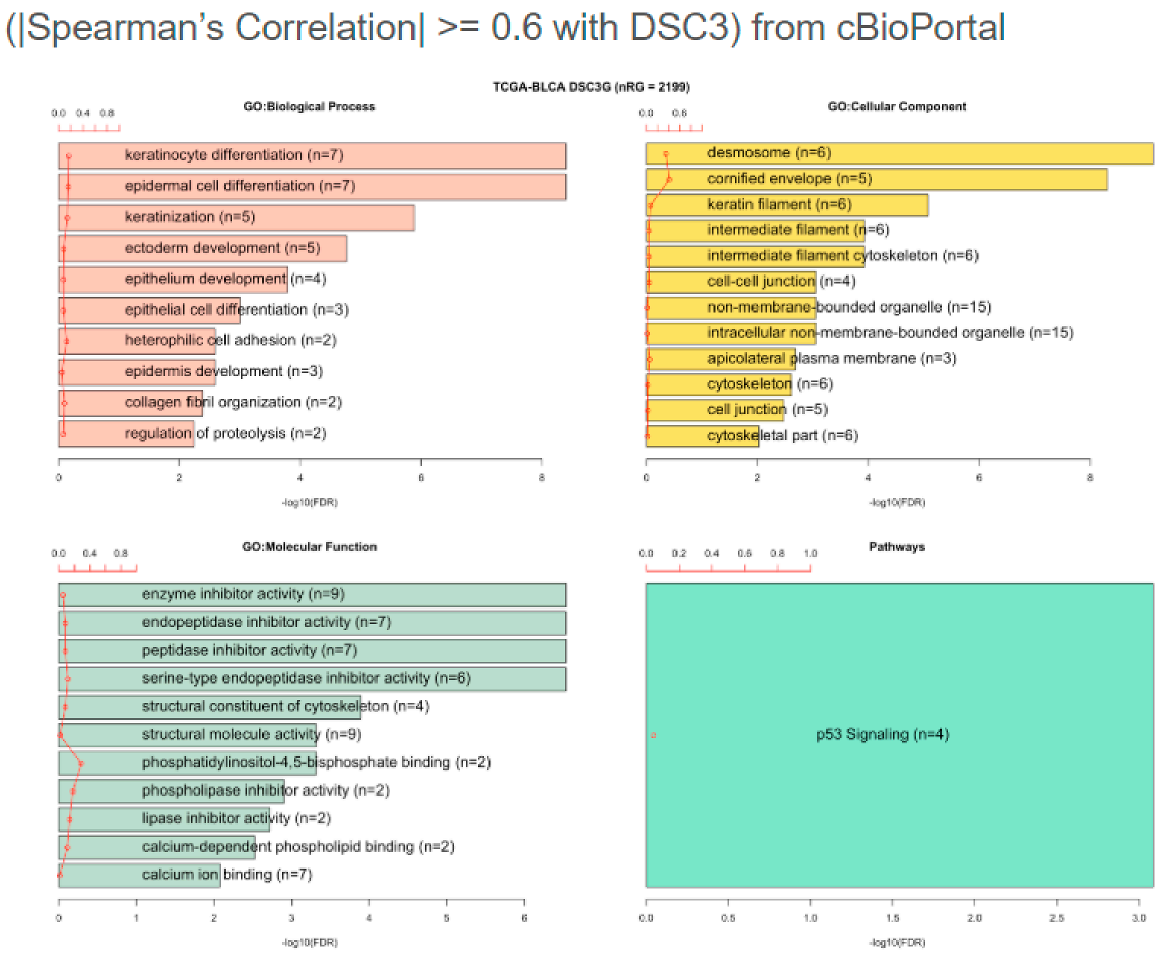

	DSC3	Non-Papillary	Papillary	MIBC
	Average	4187.8	1956.8	3304.8
	UQ	14316.2	7080.9	11502.9
	LQ	9.6	2.8	5.8

Compared to the group with lower DSC3(LQ) , group with higher DSC3 (Upper quartile) has decreased survival. (HR= 2.14; 95%CI 1.42-3.23, p=0.0002). KM plotter also provides information about impact of enrichment or decrease in immune parameters on survival. Enrichment of CD8 and NK cells is associated with further decrease in survival in DSC3 UQ group compared to DSC3 LQ group with HR=2.62 (95% CI 1.49-4.63; p= 0.00056) for NK cell enrichment and HR= 2.88 (95% CI 1.69-4.92 ;p=0.000051 for CD8 cell enrichment). However, survival became identical in both groups on decrease in NK cells and CD8 cells. Enrichment of NK cells and CD8 cells is associated with further improvement in HR in

favour of a group with lower DSC3. However, decrease in NK cells and CD8 cells is associated with identical HR.

Better survival observed in DSC3 low group compared to DSC3 high group is improved further on enrichment of NK cells and CD8 cells and disappears on their decrease.
